# Different Types of Dietary Fibers Trigger Specific Alterations in Composition and Predicted Functions of Colonic Bacterial Communities in BALB/c Mice

**DOI:** 10.3389/fmicb.2017.00966

**Published:** 2017-05-30

**Authors:** Yuheng Luo, Ling Zhang, Hua Li, Hauke Smidt, André-Denis G. Wright, Keying Zhang, Xuemei Ding, Qiufeng Zeng, Shiping Bai, Jianping Wang, Jian Li, Ping Zheng, Gang Tian, Jingyi Cai, Daiwen Chen

**Affiliations:** ^1^Key Laboratory for Animal Disease-Resistance Nutrition of China, Ministry of Education, Animal Nutrition Institute, Sichuan Agricultural UniversityChengdu, China; ^2^Laboratory of Microbiology, Wageningen UniversityWageningen, Netherlands; ^3^School of Animal and Comparative Biomedical Sciences, University of Arizona, TucsonAZ, United States

**Keywords:** dietary fibers, colonic bacterial community, 16S high throughput sequencing, PICRUSt predicted functions, BALB/c mice

## Abstract

Soluble dietary fibers (SDF) are fermented more than insoluble dietary fibers (IDF), but their effect on colonic bacterial community structure and function remains unclear. Thus, bacterial community composition and function in the colon of BALB/c mice (*n* = 7) fed with a high level (approximately 20%) of typical SDF, oat-derived β-glucan (G), microcrystalline cellulose (M) as IDF, or their mixture (GM), were compared. Mice in group G showed a lowest average feed intake (*p* < 0.05) but no change on the average body weight gain (*p* > 0.05) compared to other groups, which may be associated with the highest concentration of colonic propionate (*p* < 0.05) in these mice. The bacterial α-diversity of group G was significantly lower than other groups (*p* < 0.01). In group G, the relative abundance of bacteria belonging to the phylum *Bacteroidetes* was significantly increased, whereas bacteria from the phylum *Firmicutes* were significantly decreased (*p* < 0.01). The core bacteria for different treatments showed distinct differences. *Bacteroides*, *Dehalobacterium*, and *Prevotella*, including known acetogens and carbohydrate fermenting organisms, were significantly increased in relative abundance in group G. In contrast, *Adlercreutzia*, *Odoribacter*, and *Coprococcus* were significantly more abundant in group M, whereas *Oscillospira*, *Desulfovibrio*, and *Ruminoccaceae*, typical hydrogenotrophs equipped with multiple carbohydrate active enzymes, were remarkably enriched in group GM (*p* < 0.05). The relative abundance of bacteria from the three classes of *Proteobacteria*, *Betaproteobacteria*, *Gammaproteobacteria* (including *Enterobacteriaceae*) and *Deltaproteobacteria*, were significantly more abundant in group G, indicating a higher ratio of conditional pathogenic bacteria in mice fed dietary β-glucan in current study. The predicted colonic microbial function showed an enrichment of “Energy metabolism” and “Carbohydrate metabolism” pathways in mice from group G and M, suggesting that the altered bacterial community in the colon of mice with the two dietary fibers probably resulted in a more efficient degradation of dietary polysaccharides. Our result suggests that the influence of dietary β-glucan (SDF) on colonic bacterial community of mice was more extensively than MCC (IDF). Co-supplementation of the two fibers may help to increase the bacterial diversity and reduce the conditional pathogens in the colon of mice.

## Introduction

The microbiota in the hindgut of humans and non-ruminant animals is highly complex and comprises many thousands of bacterial species (Arumugam et al., 2011; Kau et al., 2011). The number of microbial gene copies is approximately 100 times more than that of the host (Bäckhed et al., 2004), and thus, the gut microbiota has been referred to as another “organ” of the animal body (O’Hara and Shanahan, 2006). The composition of the gut microbial community can be strongly affected by many factors such as diet (Walker et al., 2011), age (Yatsunenko et al., 2012), antibiotic administration (Relman, 2012), host genotype (Walker et al., 2011) and immunological response (Hooper et al., 2012), or infection with pathogenic organisms (Kau et al., 2011). The change of dietary components can quickly lead to a change in the composition of the microbiota (Flint et al., 2012). Of those dietary components, dietary fiber has been proven to significantly alter the composition of the intestinal microbiota (Lattimer and Haub, 2010).

There are up to 1,000 different species of bacteria that reside in the colon with microbial communities comprising approximately 10^11^–10^12^ cells/g of contents (Gibson et al., 2010). The colonic environment is favorable for bacterial growth due to its slow transit time, readily available nutrients, and favorable *pH* (Cummings and Macfarlane, 1991). Dietary fibers that escape the digestion in the small intestine are passing largely intact into the colon where they increase viscosity and bulking of the fecal matter (Lattimer and Haub, 2010) and can be degraded by microbiota in the hindgut of animals (El Aidy et al., 2013). For example, a recent study showed that ingestion of a diet low in fermentable carbohydrates resulted in lower abundance of *Firmicutes*, including the families *Clostridiaceae*, *Lachnospiraceae*, and *Ruminococcaceae* (Belcheva et al., 2014). 16S rRNA gene sequence analysis revealed that the fecal microbiota from rural African children consuming a plant-based agrarian diet rich in fruit and legume fiber (12.6 g/14.2 g total fiber) was enriched with bacteria belonging to the phyla *Actinobacteria* and *Bacteroidetes*, with unique bacteria belonging to the genera *Prevotella* and *Xylanibacter*, but had lower levels of *Firmicutes* compared to European (EU) children. Whereas, pathobionts belonging to the genera *Shigella* and *Escherichia* were more abundant in EU samples (De Filippo et al., 2010). Opposite results were also reported in another study, where a diet low in fat and high in dietary fiber was associated with increased relative abundance of bacteria belonging to the phylum *Firmicutes*, whereas a diet high in fat was associated with increased relative abundance of members of the *Actinobacteria* and genus *Bacteroides* (Wu et al., 2011). In humanized gnotobiotic mice, consumption of a Western diet was associated with an increase in the relative abundance of *Firmicutes*, the *Bacilli* (mainly *Enterococcus*) and a decrease in the proportional representation of members of the *Bacteroidetes* (Turnbaugh et al., 2009). In another study, Il10^-/-^ mice, fed a low-fat diet promoted more *Firmicutes* bacteria (Devkota et al., 2012). In humans and CEABAC10 mice, the change of dietary composition had different influence on the abundance of bacteria belonging to the phyla *Firmicutes* and *Bacteroidetes* (David et al., 2014; Martinez-Medina et al., 2014). Taken together, these results reinforce that ingestion of dietary fiber alters the bacterial community in the hindgut of non-ruminant animals.

Several recent studies revealed the effects of different fibers on colonic microbiota composition of host. Wheat bran promoted the enrichment of butyrate-producing bacteria releasing ferulic acid in the colon of human *in vitro* (Duncan et al., 2016). In diet-induced obese C57BL/6J mice, non-digestible fructans reduced their fecal bacterial diversity and increased the relative abundance of *Actinobacteria* as well as *Verrucomicrobia* compared with cellulose (Liu et al., 2016). Another study in mice fed a high-fat diet showed that the supplement of maize-derived non-digestible feruloylated oligo- and polysaccharides increased the abundance of *Blautia* and *Akkermansia* in the cecum of some mice, which was associated with reductions in body and adipose tissue weights (Yang et al., 2016). The results suggest a relationship between the type of dietary fiber and the microbial community in the hindgut of human and mice. Although dietary fiber has been classified according to its solubility in an attempt to relate physiological effects to chemical types of fiber (Marlett et al., 2002), it is recognized that additional properties of fiber, such as viscosity and fermentability, may be more important characteristics in terms of physiological benefits (Slavin et al., 2009). Generally, it is recognized that soluble dietary fibers (SDF) are fermented more and have a higher viscosity than insoluble dietary fibers (IDF), although not all SDF are viscous and some IDF may be well fermented (Slavin, 2013). However, very little is known about the *in vivo* fermentation of the two types of fibers by the colonic bacteria. Whether there are bacteria that specifically utilize SDF and IDF, the influence of single or mixed types of dietary fiber, and the impact of different types of dietary fibers on colonic microbial functions, host health, and metabolism still remain unclear.

In the present study, oat-derived β-glucan (Andersson et al., 2004) as a typical fermentable SDF fiber, and microcrystalline cellulose (MCC) (Pandey, 1999) as a typical difficult to degrade IDF (Slavin, 2013), were selected as supplementary dietary fibers. Oat β-glucan is recognized as a linear polymer of D-glucose bonded by β-(1→4) and β-(1→3) glucosidic linkage (Wang and Ellis, 2014), which is different from the D-glucose units of cellulose linearly chained via β-1,4 glycosidic linkages (Bauer et al., 1999). The exposed reducing ends of oat β-glucan molecular probably makes it soluble rather than cellulose which doesn’t have the available reducing ends. Fibers were fed to BALB/c mice for a period of 21 days, and colonic bacterial community composition and derived functions were evaluated. Notablely, unlike human or most of the non-ruminants, mice are coprophagic animals, which may influence the digestion of nutrients (Jensen et al., 2013). Thus although the results shown here may not be directly applicable to other animals, they still could potentially provide some new leads toward understanding the microbial groups specifically utilizing IDF and SDF in the hindgut of non-ruminant animals and humans.

## Materials and Methods

All experimental procedures and animal care were performed in accordance with the Guide for the Care and Use of Laboratory Animals prepared by the Institutional Animal Care and Use Committee of Sichuan Agricultural University, and all animal protocols were approved by the Animal Care and Use Committee of Sichuan Agricultural University under permit number DKY-B20131704.

### Experimental Design and Sample Collection

A total of 28 healthy male BALB/c mice at 8 weeks old were selected as experimental animals, and assigned randomly to four experimental diets based on their body weight (BW). Mice in the control group were fed a standard mouse diet formulated to meet the nutrient requirements of the American Institute of Nutrition Rodent Diets (AIN-93). Mice in the remaining three groups were fed either a diet with 28% (of total dietary ingredients) β-glucan from oat (*Arrhenatherum elatius*) (group G), 20% MCC (group M), and a mixture of β-glucan (14%) and MCC (10%) (group GM), respectively, based on the control diet. The concentrations of principal nutrients, such as lipid, protein, amino acids, minerals and vitamins of the four treatments were kept equivalent to a maximum limit (Supplementary Table S1). The β-glucan and MCC were supplied by Ci Yuan Biotechnology Co., Ltd (Shanxi, China), and the measured concentrations of β-glucan and MCC in raw ingredients were 71.2 and 98.5%, respectively. The particle size of oat-β-glucan and MCC used in current study is 108 and 150 μm, respectively. Final calculated concentrations of β-glucan in the diet of group G and GM were 19.6 and 9.8%, respectively. The calculated concentrations of MCC in the diet of groups M and GM were 19.8 and 9.9%, respectively. The concentration of SDF in the diets for the control, G, M, and GM groups were measured as 1.5, 10.1, 1.6, and 2.5 (g/100g diet), respectively, while the concentration of IDF for the four groups was 14.3, 10.9, 30.3, and 24.6 (g/100 g diet), respectively. The initial BW of the mice in the four groups (seven mice for each group, one cage for each individual) was 17.90 ± 1.07 (control), 18.20 ± 0.88 (group G), 17.60 ± 1.04 (group M) and17.40 ± 1.15 (group GM) (*p* = 0.51), respectively. The degree of purity of β-glucan and MCC and the concentrations of SDF and IDF in the diets were measured by Scist Testing Institute Co., Ltd (Qingdao, China). The concentration of β-glucan in feed sample was detected using congo red staining method. Congo red solution was prepared by dissolving 0.200 g of congo red into 0.1 mol/L phosphate buffer (*pH* 8.0) to produce 200 mL. Precisely, 0.0100 g standard β-glucan was weighed and dissolved into deionized water at 70°C. The solution was diluted to a concentration of 100 μg/mL for standard curve. Different volumes of standard solution, 0.0, 0.2, 0.6, 0.8, and 1.0 mL, were added into 10-mL colorimetric tubes and then deionized water was added into each tube to 2.0 mL, respectively. A volume of 4.0 mL cango red solution was then added into each tube, mixed and reacted at 20°C for 30 min. The absorbance of solution in each tube was detected with UV-9100 ultraviolet spectrophotometer (Beijing Persee, China) under 550 nm of wavelength. Standard curve was calculated by setting the concentration as horizontal axis and optical density value as vertical axis. The concentration of SDF and IDF was detected by following the determination of dietary fiber issued by Association of Official Analytical Chemists (AOAC International, 1996; Lee et al., 1992). The animals and the standard diet for mice were supplied by Dossy Experimental Animals Co., Ltd (Chengdu, China). For normalization and the adaptation to the environment, a pre-experiment lasted 7 days was carried out with the same diets using in formal experiment. All of the animals consumed the diets *ad libitum* and had free access to water as in formal experiment. The formal experiment lasted 21 days. During the whole experimental period, room temperature (25 ± 1.5°C) and standard humidity (70%) were maintained. Mice consumed the diets *ad libitum* and had free access to water. Feed intake of each mouse was recorded daily. BW of each mouse was recorded every 3 days. At the end of the experiment, all animals were sacrificed. Approximately 4 g of colonic digesta from each mouse was collected, kept in sterile tubes (2 ml), and immediately frozen at -80°C for genomic DNA extraction and the analysis of bacterial community.

### DNA Extraction and Sequencing

Nucleic acids for each sample was extracted immediately when the animal experiment was finished. A total of 0.04 to 0.06 g of each colonic digesta sample was weighed for DNA extraction based on the bead-beating method (Zoetendal et al., 1998). The extracted genomic DNA were stored at -80°C until further analysis. PCR amplifications were conducted with the barcoded primer pair 343f/806r set that amplifies the V3–V4 fragments of the 16S rRNA gene (343F: TACGGRAGGCAGCAG, 806R: GGACTACHVGGGTWTCTAAT). The PCR reaction (25 μl) system included 10 ng of total DNA, 1 Unit of 1× Taq buffer (Takara, Japan), 0.2 mM of each dNTP and 0.4 μM of each primer. Amplification condition consisted of an initial denaturation step of 94°C for 5 min, followed by 35 cycles of 94°C for 30 s, 56°C for 30 s, and 68°C for 45 s with a final extension at 68°C for 5 min. The PCR products (approximately 290 bp) were separated by electrophoresis in agarose gels (1.5%, w/v), purified with SanPrep DNA Gel Extraction Kit (Sangon Biotech, Shanghai, China). All PCR products were quantified with Nanodrop 2000 (Thermo Scientific, United States), pooled together with equal molar amount from each sample. The purified library was diluted, denatured, re-diluted, mixed with PhiX (equal to 30% of final DNA amount) as described in the Illumina library preparation protocols, and then applied to an Illumina Miseq system for sequencing. The sequences with high quality (max-length < 571 bp, min-length > 285 bp, average length of the effective sequences reads was 407 bp, without ambiguous base ‘N,’ and average base quality score > 30) were used for downstream analysis. A total number of 3,994,666 of V3–V4 16S rRNA valid sequences reads were obtained from 28 samples, with an average of 88,770 effective sequences reads for each sample (the minimum of one sample was 45,242 and the maximum was 122,647). All reads were deposited in the National Center for Biotechnology Information (NCBI) and can be accessed in the Short Read Archive (SRA) under accession number SRP065932.

### Bioinformatics Analysis

Pairs of reads from the original DNA fragments were merged by using FLASH (Magoč and Salzberg, 2011) (Fast Length Adjustment of SHort reads), a very fast and accurate software tool which is designed to merge pairs of reads when the original DNA fragments are shorter than twice the length of reads. Sequencing reads was assigned to each sample according to the unique barcode of each sample. Chimeric sequences were removed using USEARCH based on the UCHIME algorithm (Edgar et al., 2011). The microbial diversity was analyzed using Quantitative Insights Into Microbial Ecology (QIIME) software (Caporaso et al., 2010) with Python scripts. Operational taxonomic units (OTUs) were picked using the *de novo* OTU picking protocol with a 97% similarity threshold. Alpha diversity was estimated based on Shannon index, Chao1 and number of observed species. Jack knifed beta diversity included both unweighted and weighted Unifrac distances calculated with 10 times subsampling, and distances were visualized by PcoA (Lozupone and Knight, 2005), and the separation was tested using R in Anosim. Taxonomic assignment of OTUs was performed by comparing sequences to the Greengenes database (gg_13_5_otus).

Mann–Whitney *U*-test was used to test for significant differences in alpha diversity, and two-sided Student’s *t*-test was used to test for significant differences in beta diversity between sample groups. Linear discriminant analysis coupled with effect size (LEfSe) was performed to identify bacterial taxa differentially represented between groups at genus or higher taxonomy levels (Segata et al., 2011). The functional profiles of microbial communities were predicted by using PICRUSt (Langille et al., 2013). Bootstrap Mann–Whitney *U*-test with 1,000 per mutations was also used to identify gene pathways or OTUs with significantly different abundance among groups. The R packages ‘Phyloseq,’ ‘biom,’ ‘pheatmap’ were used for data analysis and plotting (McDonald et al., 2012; McMurdie and Holmes, 2013).

### Real-Time PCR Analysis of Bacterial Groups

The copy numbers of *Firmicutes* (Guo et al., 2008), *Bacteroidetes* (Guo et al., 2008), *Desulfovibrio desulfuricans* (Rinttilä et al., 2004), *Enterobacteriaceae* family (Bartosch et al., 2004), γ-*Proteobacteria* (De Gregoris et al., 2011), β-*Proteobacteria* (Fierer et al., 2005), *Ruminococcaceae* (Garcia-Mazcorro et al., 2012), *Prevotella* (Larsen et al., 2010), Methanogens (Denman et al., 2007), and sulfate reducing bacteria (SRB) (Kondo et al., 2004) in the colonic samples were quantified by real-time PCR on an Bio-Rad CFX-96 real time system (Bio-Rad, United States) using SYBR Green as the fluorescent dye. The primers and program of real-time PCR reaction for each bacteria was followed as the methods mentioned in the references displayed above. A reaction mixture (25 μl) consisted of 12.5 μl of IQ SYBR Green Supermix (Bio-Rad, United States), 0.2 μM of each primer set and 5 μl of the template DNA. The amount of DNA in each sample was determined in triplicate, and the mean values were calculated. To generate the standard curve for each bacterial group, the amplicons of corresponding endpoint PCR reaction performed on genomic DNA with the group-specific primer sets were first quantified (NanoDrop 2000, United States) and purified (Cloning Enhancer Kit, TaKaRa, Japan), and then serially diluted to be used as templates to generate the corresponding standard curve.

### Detection of SCFAs Concentrations

The concentrations of main SCFAs (acetate, propionate and buyterate) in the colonic digesta samples were determined using a gas chromatograph (GC) (GC-14B, Shimadzu, Japan; Capillary Column: 30 m × 0.32 mm × 0.25 μm film thickness) (Franklin et al., 2002). A total of 1 gram of digesta sample was thawed and suspended in 2 ml of distilled water and vortexed. Each sample was centrifuged (12,000 *g*) at 4°C for 10 min. The supernatant (1 ml) was then transferred into a 2-ml centrifuge tube and mixed with 0.2 ml metaphosphoric acid, and 1 μl of the supernatant was analyzed using the GC with a flame ionization detector and an oven temperature of 100–150°C (N_2_ as the carrier gas at 1.8 ml/min). The minimal measminimal detectable limit for each SCFA was 0.1 mmol/l (Luo et al., 2015).

### Statistical Analysis

Differences in the relative abundance of certain bacterial phyla or genera, average feed intake, BW gain, the numbers of bacterial groups and the concentrations of SCFAs among treatments were analyzed with one-way ANOVA using the statistical software SPSS 16.0. Differences were considered significant when *p* < 0.05.

## Results

### Feed Intake, Body Weight Gain of Mice and SCFAs Concentrations of Digesta Samples in Different Groups

The differences of average weight gain (AWG) of mice in the four groups were only found before day 12 (**Table 1** and Supplementary Figure S1). In the first period (1–3 days), the AWG of mice in group G showed significantly lower than group C and M (*p* < 0.05). From days 4 to 6, the AWG of mice in group M was significantly lower than group GM (*p* < 0.05). From days 7 to 9, the AWG of mice in group G was observed significantly higher than group C and GM (*p* < 0.05). From days 10 to 12, the AWG of mice in group G showed significantly higher than group C and M (*p* < 0.05), while no significant differences were found in AWG of mice in the four groups from days 1 to 21 (*p* > 0.05). The differences of average feed intake (ADFI) of mice in the four groups were observed during the whole experiment (**Table 1** and Supplementary Figure S1A). The difference of ADFI of mice in the four groups in each period was also showed in **Table 1**. For whole period (1–21 days), the ADFI showed significantly different among the four groups (*p* < 0.001, **Table 1** and Supplementary Figure S1B).

**Table 1:** Average daily feed intake (ADFI) and average weight gain (AWG) of mice in the four different treatments.

	Group	1–3 days	4–6 days	7–9 days	10–12 days	13–16 days	17–21 days	1–21 days
**AWG g**	C	1.36 ± 0.52^a^	0.73 ± 0.55 ^a^ ^b^	- 0.07 ± 0.35 ^b^	- 0.60 ± 0.35 ^b^	1.19 ± 0.24	- 0.41 ± 0.35	2.19 ± 0.75
	G	- 0.56 ± 0.56 ^b^	0.36 ± 0.64 ^a^ ^b^	1.20 ± 0.43 ^a^	0.84 ± 0.31 ^a^	0.90 ± 0.38	- 0.37 ± 0.32	2.37 ± 0.32
	M	1.81 ± 0.24 ^a^	- 0.53 ± 0.44 ^b^	0.34 ± 0.47 ^a^ ^b^	- 0.60 ± 0.56 ^b^	1.17 ± 0.73	- 0.79 ± 0.55	1.41 ± 0.62
	GM	0.84 ± 0.57 ^a^ ^b^	1.53 ± 0.68 ^a^	- 0.29 ± 0.23 ^b^	0.61 ± 0.35 ^a^ ^b^	0.34 ± 0.28	- 1.04 ± 0.47	2.00 ± 0.65
	*P*-value	0.014	0.123	0.053	0.027	0.503	0.655	0.705
**ADFI g/d**	C	6.03 ± 0.57 ^a^	5.89 ± 0.40 ^a^	3.70 ± 0.26 ^a^	4.49 ± 0.41 ^a^	5.90 ± 0.21 ^a^	5.51 ± 0.44 ^a^	5.33 ± 0.18 ^a^
	G	2.74 ± 0.40 ^c^	3.68 ± 0.42 ^c^	3.03 ± 0.19 ^b^	3.72 ± 0.22 ^ab^	3.41 ± 0.24 ^c^	3.18 ± 0.28 ^b^	3.42 ± 0.12 ^c^
	M	4.61 ± 0.31 ^b^	5.04 ± 0.51 ^ab^	3.31 ± 0.20 ^ab^	3.52 ± 0.25 ^b^	4.51 ± 0.39 ^b^	5.71 ± 0.25 ^a^	4.63 ± 0.18 ^b^
	GM	3.71 ± 0.48 ^bc^	4.50 ± 0.36 ^bc^	3.10 ± 0.17 ^ab^	3.57 ± 0.17 ^b^	4.89 ± 0.19 ^b^	4.77 ± 0.37 ^a^	4.19 ± 0.30 ^b^
	*P*-value	0.000	0.009	0.117	0.074	0.000	0.000	0.001

*The variant alphabetical superscript in the same column indicates significant difference when *p* < 0.05, *n* = 7. Data were shown as Mean ± Standard Errors. C, control; G, glucan; M, MCC; GM, glucan and MCC*.

The concentration of acetate and butyrate and the ratio of acetate to propionate showed no difference among the four groups (*p* > 0.05), while the concentration of propionate was significantly higher in group G and GM compared to group C and M (*p* < 0.05, **Table 2**).

**Table 2:** Concentrations of acetate, butyrate and propionate in the colonic digesta of mice in the four groups.

Item	C	G	M	GM	*P*-value
Acetate μmol/g	17.72 ± 4.35	29.28 ± 13.65	17.18 ± 6.75	27.01 ± 13.35	0.096
Propionate μmol/g	2.64 ± 1.02 ^b^	4.61 ± 2.41 ^a^	2.32 ± 0.67 ^b^	4.51 ± 1.50 ^a^	0.019
Butyrate μmol/g	1.41 ± 0.98	0.77 ± 0.55	2.03 ± 2.27	1.02 ± 0.47	0.328
Acetate/Propionate	7.13 ± 1.62	6.51 ± 1.57	7.47 ± 2.52	6.35 ± 3.05	0.784

*The variant alphabetical superscript in the same row indicates significant difference when *p* < 0.05, *n* = 7. Data were shown as Mean ± Standard Deviation. C, control; G, glucan; M, MCC; GM, glucan and MCC*.

### Metadata and Sequence Analysis

After assigning OTU and chimera checking, sequencing for 28 samples yielded 2,296,025 sequences with an average of 82,001 ± 13,719 sequences per sample. All reads were assigned to 4,368 non-singleton OTUs, which could be assigned to a total of 25 bacterial phyla and 275 bacterial genera. Each sample had 753 ± 205 OTUs on average (Supplementary Table S2).

### The Microbial Diversity in the Colonic Samples of Different Treatments

**FIGURE 1:**
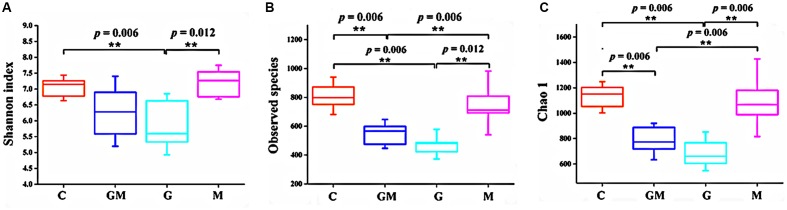
Differences on bacterial community diversity and richness among the four treatments. **(A)** Shannon index; **(B)** Observed OTUs; **(C)** Chao1 index. Differences were considered significant when *p* < 0.05, ^∗^*p* < 0.05, ^∗∗^*p* < 0.01. C, control; G, glucan; M, MCC; GM, glucan and MCC.

Three alpha diversity measures were calculated including Shannon’s diversity index, observed species (observed OTUs), and Chao1 (estimated number of OTUs). The Shannon index of group G was significantly lower than that of the control and M groups (*p* < 0.01, **Figure 1A**). Observed and expected bacterial richness were significantly lower in groups G and GM than those of the control and M groups (*p* < 0.01, **Figures 1B,C**).

### Composition of Bacterial Communities in the Colonic Digesta of Mice in Different Groups

**FIGURE 2:**
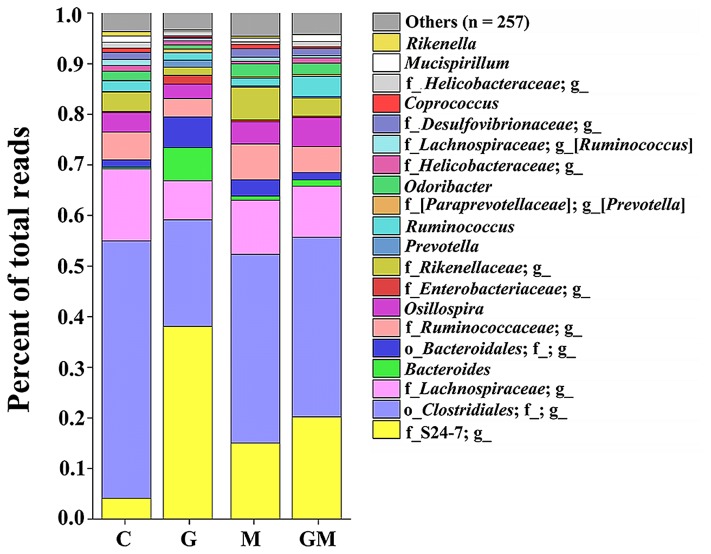
Bacterial composition in the colonic digesta of mice from the four treatments. Each bar represents the average relative abundance of each bacterial taxon within a group. The top 20 most abundant taxa are shown. These taxa were observed with high relative abundance in the four groups, and comprise a relative abundance ranging from 95.4 to 96.7%. g, genus level; f, family level, o, order level. The underline followed by no annotation means no known taxonomic level was classified. C, control; G, glucan; M, MCC; GM, glucan and MCC.

A total of 25 phyla were detected in the colonic digesta of the experimental mice. Of these 25, six phyla (*Actinobacteria*, *Bacteroidetes*, *Deferribacteres*, *Firmicutes*, *Proteobacteria*, and *Tenericutes*) were the most predominant, each comprising more than 0.1% of the total reads per group (**Table 3** and Supplementary Figure S2). The relative abundance of bacteria from the phyla *Bacteroidetes* and *Firmicutes* was significantly different between groups (*p* < 0.001). The relative abundance of bacteria from the phylum *Bacteroidetes* in group G was significantly higher than that in the control, M and GM groups (*p* < 0.01), whereas its relative abundance (12.9 ± 4.2%) was lowest in the control group and significantly lower than that in groups M and GM (*p* = 0.025, 0.020, respectively) (**Table 3**). The relative abundance of bacteria from the phylum *Firmicutes* was highest in the control group (80.1 ± 4.8%). In contrast, relative abundance of *Firmicutes* was significantly lower in group G (38.7 ± 14.8%), than that in groups M and GM (*p* = 0.001, 0.002, respectively) (**Figure 2**, **Table 3**, and Supplementary Figure S3). In addition to the two predominant phyla, *Proteobacteria* was observed as the third most predominant phylum in the colonic samples of the all four groups with no significant differences between groups (*p* > 0.05). All five classes of *Proteobacteria* (*Alpha*-, *Beta*-, *Delta*-, *Epsilon*-, and *Gamma-proteobacteria*) were found in all of the samples. Relative abundance of *Betaproteobacteria* and *Gammaproteobacteria* from samples in group G was significantly higher than that in the control, M and GM groups (*p* = 0.015, 0.005, 0.037 and 0.023, 0.034, 0.018, respectively) (Supplementary Figure S4), while relative abundance of *Deltaproteobacteria* in group G was significantly lower than that in the other groups (*p* = 0.013, 0.002, 0.044, respectively) (Supplementary Figure S4). At the family level, the relative abundance of the *Enterobacteriaceae* was significantly higher in group G than in the other three groups (*p* ≤ 0.016).

**Table 3:** Relative abundance (%) of main bacterial phyla (more than 0.1%) in the colon of mice in different treatments.

Phylum	C	G	M	GM	*P*-value
*Actinobacteria*	0.12 ± 0.06	0.23 ± 0.23	0.20 ± 0.23	0.10 ± 0.08	0.41
*Bacteroidetes^∗∗^*	12.93 ± 4.18	55.41 ± 15.70	29.36 ± 14.72	30.07 ± 13.38	0.00
*Deferribacteres*	1.23 ± 0.88	0.23 ± 0.21	0.75 ± 1.20	1.32 ± 1.47	0.22
*Firmicutes^∗∗^*	80.09 ± 4.77	38.69 ± 14.78	64.10 ± 13.33	62.35 ± 15.50	0.00
*Proteobacteria*	5.08 ± 1.72	4.92 ± 2.67	5.10 ± 1.61	5.33 ± 2.89	0.99
*Tenericutes*	0.35 ± 0.29	0.27 ± 0.57	0.18 ± 0.19	0.63 ± 1.55	0.77

*The difference in relative abundance of bacterial phyla among the four groups was analyzed with One-Way ANOVA using the statistical software SPSS 16.0. The background color of each cell indicates relative abundance of each phylum with red and yellow indicating highest and lowest values. Only phyla with a relative abundance of more than 0.1% are shown. Data were shown as Mean ± Standard Deviation. C, control; G, glucan; M, MCC; GM, glucan and MCC. Differences were considered significant when *p* < 0.05, ^∗∗^*p* < 0.01*.

**FIGURE 3:**
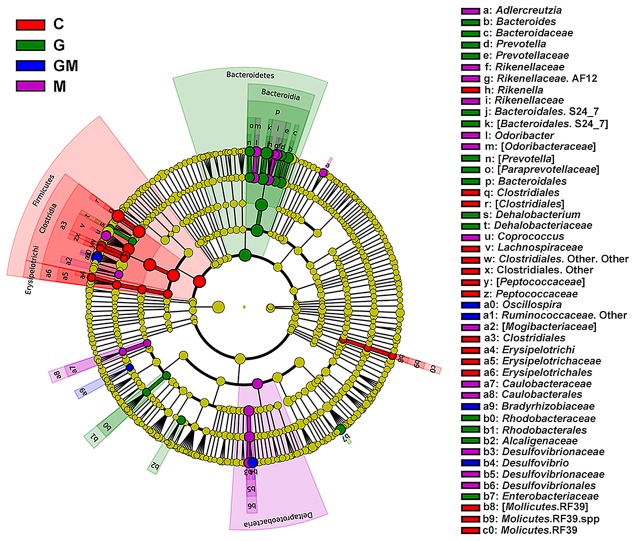
Histogram of the linear discriminant analysis (LDA) scores computed for bacterial taxa differentially abundant among the four groups using default parameters. Six levels of the biomarkers (from phylum to genus) were shown in this figure. The circles radiating from inner to outside mean levels from phylum to genus. Small spots on each circle mean sub-classifications on the corresponding level, and the size of each spot is in proportion to the corresponding sub-classification. Sub-classifications with no differences among the treatments are colored with yellow, while biomarkers in each treatment are colored with different colors. A total of 53 differentially abundant bacterial taxa were detected. Of those, 17 taxa were significantly overrepresented in the colon of mice fed with oat-derived β-glucan (green), 14 taxa were overrepresented in mice fed with MCC (purple), 4 taxa were overrepresented in mice fed the mixture of the two fibers (blue), and 14 taxa were overrepresented in controlled mice (red). C, control; G, glucan; M, MCC; GM, glucan and MCC.

Linear discriminant analysis (LDA) and LDA coupled with effect size (LefSe) showed that a total of 53 bacterial taxa were different in relative abundance (α = 0.01, LDA score > 3.0) among the four groups (**Figure 3** and Supplementary Figure S5). A total of 17 taxa were significantly more abundant in group G than in the other groups (e.g., *Bacteroides* spp., *Dehalobacterium* spp., *Prevotella* spp., *p* < 0.05), 14 taxa were significantly more abundant in group M (e.g., *Adlercreutzia* spp., *Odoribacter* spp., *Coprococcus* spp., AF12, *p* < 0.05), 18 taxa were significantly more abundant in the control group (e.g., *Rikenella* spp., *p* < 0.05), while only four taxa were significantly more abundant in the GM group (e.g., *Oscillospira* spp., *Desulfovibrio* spp., *Ruminoccaceae*, *p* < 0.05) (**Figure 3** and Supplementary Figures S3, S6). From these 53 taxa, those had a relative abundance of at least 0.01% and occurred in at least one of the treatments were further tested using analysis of variance (ANOVA) (Supplementary Table S3).

### Core Colonic Bacterial Communities of Mice in Different Treatments

**FIGURE 4:**
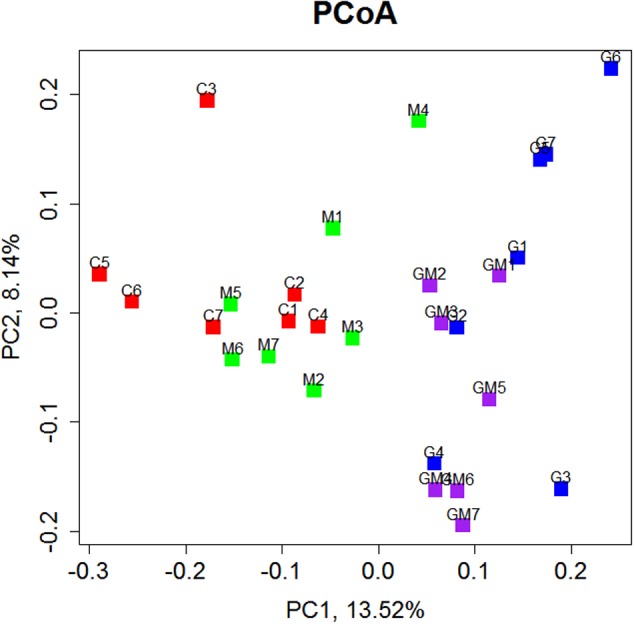
Principal Coordinate Analysis (PCoA) of bacterial community structures of the colonic microbiota of four experimental groups, each represented by one color. PCoA shows distinct bacterial communities for the four different treatments (*p* = 0.001). C, control; G, glucan; M, MCC; GM, glucan and MCC.

The relationships among the colonic bacterial community structures of the four groups were examined by using Principal Coordinates Analysis (PCoA) based on an unweighted UniFrac distance matrix (**Figure 4**), indicating that there was no significant separation of colonical microbiome between the control and group M, whereas the control and GM group, control and G group, and groups M and G can be significantly separated (*R* = 0.379, *p* = 0.001, Supplementary Figure S7).

**FIGURE 5:**
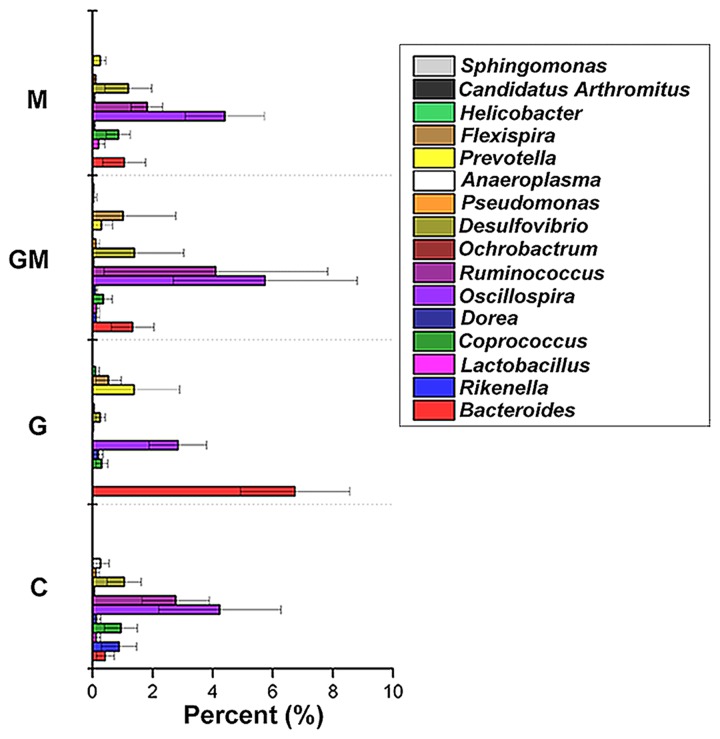
Core bacterial genera in the colon of mice in the four different groups. To test for evidence of a core bacterial community in the colonic samples, the compute_core_microbiome function within QIIME was used, requiring the core OTUs to be present in at least 90% of the samples as based on the non-rarefied data set. C, control; G, glucan; M, MCC; GM, glucan and MCC.

The core bacterial community for each treatment was analyzed based on the data from the seven individuals in the treatment, and was defined as those taxa found in 90% of the colonic samples for each treatment. For group G and the controls, the core community consisted of 11 OTUs, and the reads of the core OTUs comprised as much as 96.6 and 95.4% of the total community, respectively. For groups GM and M, 10 and 14 OTUs were found, respectively, as the core bacterial community, and the core community comprised 96.9 and 95.4% of the total community, respectively. Within the core bacterial community, *Bacteroides* (41.7%), *Oscillospira* (19.9%), *Prevotella* (10.7%), and *Ruminococcus* (10.0%) were identified as the four most predominant genera in the colon of mice fed the glucan-diet. These four genera comprised approximately 82.3% of the core community (**Figure 5**). For mice fed the MCC-diet, *Oscillospira* (39.7%), *Ruminococcus* (13.6%), *Desulfovibrio* (11.1%), *Coprococcus* (7.5%), and *Bacteroides* (7.1%) were the top five genera, comprising 79.0% of the bacterial community. The genera *Oscillospira* (35.2%), *Ruminococcus* (26.4%), *Desulfovibrio* (8.9%), *Bacteroides* (8.0%), and *Flexispira* (6.2%) comprised the main core of bacteria in the colon of mice fed with the mixed (GM) diet, accounting for 84.7% of the core microbiota (**Figure 5**). *Oscillospira* (32.7%), *Ruminococcus* (18.8%), *Flexispira* (10.2%), *Desulfovibrio* (8.6%), *Coprococcus* (7.4%), and *Rikenella* (6.8%) were the six dominant genera in the colonic samples of the control group, and comprised approximately 84.5% of the total core community (**Figure 5**).

### Quantification of Specific Bacterial Groups in the Four Treatments Using Q-PCR

As shown in **Table 4**, the numbers of *Enterobacteriaceae*, *Prevotella*, and β-*Proteobacteria* in the colonic samples from group G were significantly higher than other groups (*p* < 0.05), while the number of *Ruminococcaceae* of group G showed significantly lower than others (*p* < 0.01). The copy number of *D. desulfuricans* showed lowest in samples from group G and highest in those from group GM (*p* < 0.01). The quantity of *Bacteroidetes* from group G were significantly higher than the control group (*p* = 0.01), while the number of *Firmicutes* were significantly higher in the controls than in the other groups (*p* < 0.01). The number of γ-*Proteobacteria* in group G was significantly higher than the M and control groups (*p* < 0.05). For the two main hydrogenotrophic bacteria, the copy number of SRB in group G was significantly lower than the other groups with a significantly higher number in the GM and M groups than the control group (*p* < 0.01), while no significant difference on the number of methanogens was found among the four groups (*p* > 0.05).

**Table 4:** The quantity of certain bacterial groups in the colonic digesta from mice in the four treatments [log_10_(copy numbers of gene)/gram of wet sample].

Bacteria	C	G	M	GM	*P*-value
*Firmicutes*	11.80 ± 0.37^a^	10.80 ± 0.42 ^b^	11.06 ± 0.31 ^bc^	11.22 ± 0.36 ^c^	0.00
*Ruminococcaceae*	10.46 ± 0.74 ^a^	9.43 ± 0.73 ^b^	10.53 ± 0.42 ^a^	10.86 ± 0.33 ^a^	0.00
*Bacteroidetes*	10.11 ± 0.55 ^a^	11.12 ± 0.56 ^b^	10.58 ± 0.54 ^ab^	10.57 ± 0.33 ^ab^	0.01
*Prevotella*	5.93 ± 0.53 ^a^	6.54 ± 0.39 ^b^	5.95 ± 0.32 ^a^	6.04 ± 0.23 ^a^	0.02
β -*Proteobacteria*	6.74 ± 0.20 ^a^	8.04 ± 0.47 ^b^	7.05 ± 0.42 ^a^	7.03 ± 0.44 ^a^	0.00
γ -*Proteobacteria*	9.15 ± 0.62 ^a^	10.13 ± 0.41 ^b^	9.30 ± 0.88 ^a^	9.70 ± 0.62 ^ab^	0.04
*Enterobacteriaceae*	7.04 ± 0.26 ^a^	8.53 ± 0.71 ^b^	7.43 ± 0.24 ^a^	7.23 ± 0.22 ^a^	0.00
SRB	8.07 ± 0.49 ^a^	7.25 ± 0.36 ^b^	9.05 ± 0.35 ^c^	8.61 ± 0.37 ^c^	0.00
*Desulfovibrio desulfuricans*	8.12 ± 0.35 ^a^	6.60 ± 0.27 ^b^	8.20 ± 0.20 ^a^	8.59 ± 0.21 ^c^	0.00
Methanogens	6.93 ± 0.21	6.98 ± 0.34	6.91 ± 0.30	6.90 ± 0.23	0.95

*Data was shown as Mean ± Standard Deviation. C, control; G, glucan; M, MCC; GM, glucan and MCC. ^a,b,c^Different letter superscripts in same column indicate significant difference (*p* < 0.05)*.

### Predicted Functional Composition of Colonic Microbial Communities of Mice in Different Treatments

**FIGURE 6:**
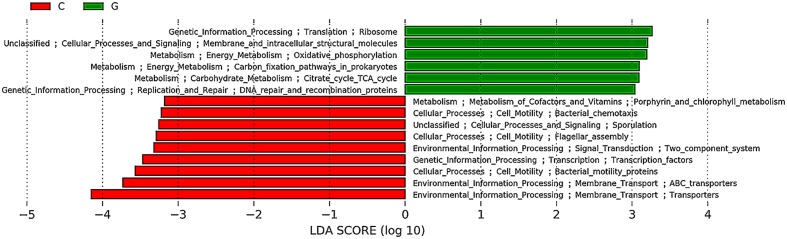
Predicted microbial pathways significantly differentiated between control and group G identified by linear discriminant analysis coupled with effect size (LEfSe) using the default parameters. C, control; G, glucan.

To investigate alterations in colonic microbial function, the software program Phylogenetic Investigation of Communities by Reconstruction of Unobserved States (PICRUSt) was applied to the 16S rRNA gene sequencing data. PICRUSt results were then analyzed using LEfSe to identify microbial functions that were significantly different in their relative abundance among groups. Of the 248 KEGG (Kyoto Encyclopedia of Genes and Genomes) pathways, a total of 15 differentially abundant bacterial functions were observed especially between the control and group G (α = 0.01, LDA score > 3.0) (**Figure 6**). Of those, three pathways on energy and carbohydrate metabolism (e.g., “Oxidative phosphorylation” and “Tricarboxylic acid cycle”), two pathways on genetic information processing (e.g., “Ribosome”), and one pathway on “Membrane and cellular structural molecules” were overrepresented in glucan-fed mice (**Figure 6**).

## Discussion

The health benefits of dietary fiber have long been appreciated. Dietary fiber has been shown to protect against development of diseases, including diabetes, cardiovascular disease, colon cancer, and obesity (Slavin, 1987; Roberfroid et al., 2010). Recent studies indicate a link between intake of dietary fibers and colonic bacterial communities in animals and humans (Chassard et al., 2010; De Filippo et al., 2010; Isken et al., 2010). However, the effect of different types of dietary fibers, especially fermentable SDF and IDF, on colonic microbial community composition and associated functions, was often ignored. Thus, in the present study, we investigated bacterial community composition using sequencing of PCR-amplified 16S rRNA gene fragments and further predicted associated microbial functions in the colon of BALB/c mice fed with diets supplemented with either oat-derived β-glucan, MCC, or the mixture of both fibers. Moreover, the changes on the relative abundance of bacteria were confirmed by the qPCR results.

In the current study, the decrease of ADFI of mice fed with β-glucan enriched diet was probably associated with the gel-forming properties (Isken et al., 2010) of SDF and increased distention (Slavin, 2013). Study on humanized mice showed that diet, such as fiber-containing diet can modify GI transit, which then affects the composition of the microbial community (Kashyap et al., 2013; Roager et al., 2016). On the other hand, the higher feed intake has been proved to increase the length of the intestine of mice (Selman et al., 2001), while the ingestion of high ratio of dietary carbohydrates may not have this affect (Shibuya et al., 2015). According to our result, the ADFI of mice in control group showed a highest intake, which may suggest a longer intestine and rapid gut throughput of this group, and in turn, affect the microbial community. As the length of intestine and transit time of the mice was not investigated in current study, further study will be needed to verify this speculation. Although the ADFI of the mice fed with glucan-containing diet was lowest for the whole experimental period, the AWG of the mice showed increased from day 7, which elicited the similar AWG between groups at the end of the experiment. This may be related to the remarkable increase of propionate in the colon of mice in group G. In some non-ruminant (Verbrugghe et al., 2012) animal and ruminants (Zhang et al., 2016), gut microbe-produced propionate can be absorbed and used by host as one of the main substrates of gluconeogenesis. A large amount of propionate production is commonly observed during the fermentation of soluble complex carbohydrates (Stevens and Hume, 1998; German et al., 2015). Although the increase of acetate in the colonic digesta of mice in group G and GM was not remarkable due to the larger individual differences (a larger sample size may have led to significant differences), the concentration of acetate in the samples in these two groups showed a substantial improvement. Acetate produced by gut microbes is one of the energy source of host (Kimura et al., 2016) as propionate, the observed higher concentration of propionate and acetate in these mice might be directly utilized by the intestinal epithelial cells as energy source of host, which needs to be further verified with highly sensitive method such as metabolomics analysis or under a larger sample size.

The α diversity index of colonic bacteria was significantly decreased in animals fed diet G as compared to animals fed the control diet, while the bacterial diversity in mice fed the MCC diet was not different from those given the control diet, which was also confirmed by the PCoA plot. No decrease was found in animals fed with diet M, whereas the mixed diet, containing lower amounts of both fibers, also led to a reduced diversity, albeit slightly less pronounced than for diet G. Although only one representative fiber per type was investigated in the present study, it is tempting to speculate based on our findings that the supplement of SDF, such as oat-derived β-glucan, but not IDF, may reduce the biodiversity of colonic microorganisms of mice. A study on rats showed that compared to non-nutritive cellulose-fed rats (56 and 56.8 g/kg diet), supplementation of high-viscosity β-glucan in the diet increased the bacterial diversity in the cecum of rats as measured by denaturing gradient gel electrophoresis (DGGE) of 16S rRNA gene fragments (Snart et al., 2006). This was not confirmed by the present study, however, this difference may be due to the high proportion of fibers (approximately 20% in each diet) in the present study.

The relationships among the colonic bacterial community structures of the four groups were examined by using PCoA based on an unweighted UniFrac distance matrix (**Figure 4**), indicating that there was no significant separation of colonical microbiome between the control and group M, while the control and GM group, control and G group, and groups M and G can be significantly separated (*p* = 0.001). At the phylum level, *Firmicutes* and *Bacteroidetes* were found as the most predominant taxa in all colonic samples, which is consistent with previous studies in humans and mice (Bäckhed et al., 2005; Tremaroli and Bäckhed, 2012; Yatsunenko et al., 2012). In the present study, the relative abundance of *Bacteroidetes* was remarkably increased in the colon of mice fed with high concentrations of β-glucan, whereas the addition of MCC led to a decrease in relative abundance of this phylum (Supplementary Figure S3). It has been reported that a diet reduced in fermentable carbohydrates decreased the relative abundance of representatives from the phylum *Firmicutes* (Belcheva et al., 2014). However, only the *Firmicutes*/*Bacteroidetes* ratio of the control group was significantly higher than other groups (*p* ≤ 0.01). The difference of *Firmicutes*/*Bacteroidetes* between group G (*p* = 0.078) and M (*p* = 0.067) was nearly significant. Our findings suggest that although the microbial community in the distal gut may be altered by dietary fiber, probably some SDF like oat-derived β-glucan, but not IDF such as MCC, might have primary impact on the community structure.

*Proteobacteria* is also a major phylum in the gut of animals. In the present study, although no change was found at the phylum level, the relative abundance of bacteria from the three classes of *Proteobacteria* were significantly changed in mice, fed with glucan-supplemented diet, but the trends were not consistent. The relative abundance of *Betaproteobacteria*, *Gammaproteobacteria*, and family *Enterobacteriaceae* within the *Gammaproteobacteria* was increased with the present of dietary β-glucan (SDF) (Supplementary Figure S4). Gut bacteria belonging to the *Beta*-and *Gammaproteobacteria* generally include the majority of potential pathogens (Hernandez-Doria and Sperandio, 2013; Cariveau et al., 2014), while the family *Enterobacteriaceae* contains Gram-negative bacteria including harmless symbionts and pathogens, such as *Escherichia coli, Salmonella*, and *Yersinia pestis* (Stecher et al., 2012). In the current study, when dietary β-glucan was increased, potential pathogens such as those in the *Enterobacteriaceae* increased, which was further confirmed by real-time PCR results. We also found that the relative abundance of bacteria belonging to the *Deltaproteobacteria* was decreased with the increase of IDF/SDF ratio (Supplementary Figure S4). A large number of bacteria in the *Deltaproteobacteria* are affiliated to the SRB (Rodionov et al., 2004; Sorokin et al., 2008), a cluster of hydrogenotrophs (Vianna et al., 2008) that can improve the fermentation efficiency of gut microorganisms by removing end products such as H_2_. MCC-degrading species were shown to produce high amounts of H_2_ from cellulose fermentation (Chassard et al., 2010). In addition, H_2_ is needed to produce propionate by certain bacteria (Hosseini et al., 2011), but the concentration of propionate was only found remarkably increased in the samples of mice fed with glucan-containing diet with a lowest quantity of SRB, which suggests that the producing of propionate but not SRB might be one of the ways to remove H_2_ by colonic bacteria of these mice. One of the main sulfate reducing product by SRB is H_2_S which is generally recognized as a signaling molecule mediating gastrointestinal secretion and intestinal peristalsis, and also associated with the inflammatory reaction of intestine (Farrugia and Szurszewski, 2014). However, as the production of H_2_S and H_2_ is very hard to monitor *in vivo* in mice, whether the increase of SRB was harmful or not to the gut health of mice cannot be confirmed in current study. According to real-time PCR results, the number of SRB was remarkably higher in groups M and GM. Considering there was no change on the methanogens, the change of SRB found in the current study may indicate that SRB were the preferred hydrogen utilizers, rather than methanogens, in the colon of mice consuming the MCC diet. However, this assumption needs to be verified by further studies.

The core microbial community was unique in mice from group G. Both *Bacteroides* and *Prevotella* spp. were found in group G, and both are able to degrade diverse plant polysaccharides, most of which are fermentable fibers and native cellulose (Chassard et al., 2010). However, cellulolytic enzymes have not yet been characterized from any human *Bacteroides* strain (Flint et al., 2012). Bacteria affiliated to *Bacteroides* spp. were also observed as a core genus in the colon of mice fed with MCC, and the glucan and MCC mixed-diet, presented lower relative abundances, whereas *Bacteroides* spp. were predominant in G-diet fed mice. This suggests these bacteria play an important role during the degradation of oat β-glucan. *Prevotella* spp. have been shown to utilize fermentable complex carbohydrates (Ivarsson et al., 2014). In people who consume more carbohydrates, especially fiber, *Prevotella* spp. Dominate (Wu et al., 2011), making up 53% of the gut bacteria of African children (De Filippo et al., 2010). In the present study, *Prevotella* was only observed as a core genus in group G mice, which confirms the studies mentioned above. However, *Prevotella* are also implicated in TMAO formation from meat carnitines, thereby exacerbating the cardiovascular outcomes of red meat consumption (Koeth et al., 2013). Notably, *Flexispira* spp., initially isolated from aborted sheep fetuses (Kirkbride et al., 1985), and formerly recognized as a genus associated with human chronic diarrhea (Archer et al., 1988), was found as a core genus in mice from the group G and GM.

Members of the acetogenic genus *Dehalobacterium* were found in samples from glucan-fed mice. In contrast, non-or moderate-acetogens and hydrogen-producing bacteria, such as *Adlercreutzia* spp. (Maruo et al., 2008), *Odoribacter* spp. (Nagai et al., 2010) and *Coprococcus* spp. (Patel et al., 1981) were found in samples from MCC-fed mice. This further confirmed that the increase of SRB in mice from groups M and GM might probably be associated with the production of H_2_ during the fermentation of MCC. In addition, bacteria with strong fiber-degradation capability, such as bacteria in the family *Ruminoccaceae*, which are well known to degrade SDF, but also IDF, with their multiple carbohydrate active enzymes (CAZy) (Flint et al., 2012), were found in samples from mice fed with glucan and MCC-mixture. This further suggests a potential specialization in ecological niches or guilds in the hindgut of mice consuming different types of dietary fibers. The predicted functional analysis further confirms this proposition.

**FIGURE 7:**
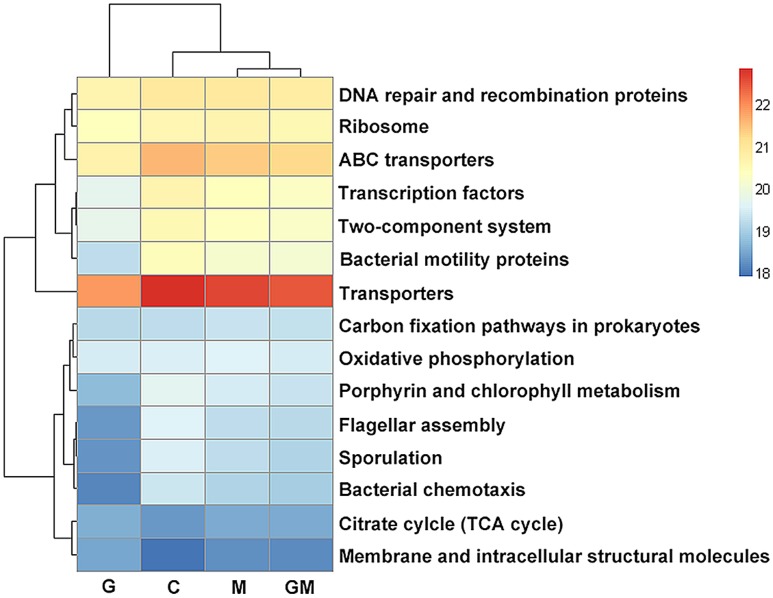
Predicted function of colonic microbiota among mice in the four treatments. The gene copy numbers of samples within the same sample group were pooled. Values of each functional gene (row) were log10 transformed. All of the three levels of KEGG pathways are shown in the heatmap. Significance of differences in gene distribution among groups was tested using bootstrap Mann–Whitney *U*-test with cutoffs of *p* < 0.01, FDR < 0.1, Mean counts > 10. Genes with significance of differences among groups from 0.01 to 0.05 were not shown. C, control; G, glucan; M, MCC; GM, glucan and MCC.

With respect to metabolic pathways, the enrichment of “Energy metabolism” and “Carbohydrate metabolism” pathways in glucan-fed mice are remarkable, indicating a high activity on carbohydrate utilization of bacteria in the colon of these mice. β-glucan has been proved as a SDF with high fermentability (Andersson et al., 2004). The supplement of oat derived β-glucan may directly improve the ability of polysaccharides degradation of colonic bacteria. This functional change is consistent with the increase of SDF degrading bacteria mentioned above, such as *Bacteroides* and *Prevotella* spp. Interestingly, the enrichment of these two types of pathways in samples from group M and GM were also found in current study (**Figure 7**), although compared with control group, the change was not significant. Our result suggests that both of SDF and IDF may increase the capability of polysaccharide utilization of colonic bacteria in mice, with a larger influence of SDF.

In summary, the high level (approximately 20%) supplement of oat-derived β-glucan into diet, rather than MCC or their mixture, remarkably decreased the bacterial α-diversity in the colon of BALB/c mice in current study. The colonic bacteria can specifically response to different types of dietary fibers, and different core bacterial species were found in the colon of mice fed with corresponding fibers. The altered bacterial community in the colon of mice with the two dietary fibers resulted in a more efficient degradation of dietary polysaccharides, which was further confirmed by the enrichment of “Energy metabolism” and “Carbohydrate metabolism” pathways in these mice. The higher concentration of colonic propionate in mice fed glucan-containing diet may be associated with the lowest ADFI and highest AWG of these mice. Although dietary β-glucan increased the relative abundance of potential pathogens belonging to *Proteobacteria*, such as *Betaproteobacteria*, *Gammaproteobacteria* (including *Enterobacteriaceae*) and *Deltaproteobacteria*, no significant change was found on the concentration of acetate and butyrate in the colonic digesta of these mice. Co-supplementation of the two fibers may be more beneficial to increase the diversity of colonic bacteria and reduce the conditional pathogens.

## Author Contributions

YL designed the experiment, wrote the paper, and had primary responsibility for the final content. LZ conducted the animal trial and data analysis. HL conducted the data analysis of sequencing. A-DW and HS helped to analyze the data and write the paper. KZ, XD, QZ, SB, and JW helped to conduct the animal trial and sample collection. JL prepared the feed and helped to conduct the animal trial. PZ, GT, JC, and DC helped to design the experiment. All authors read and approved the final manuscript.

## Conflict of Interest Statement

The authors declare that the research was conducted in the absence of any commercial or financial relationships that could be construed as a potential conflict of interest.
