# Supplementary material for: Different Types of Dietary Fibers Trigger Specific Alterations in Composition and Predicted Functions of Colonic Bacterial Communities in BALB/c Mice
Source: Front Microbiol. 2017 May 30;8:966. doi: 10.3389/fmicb.2017.00966 (PMC5447771; doi:10.3389/fmicb.2017.00966)
Supplement: Supplementary file 1 [file Presentation_1.pdf]

Supporting Informations

Journal name: Frontiers in Microbiology

Manuscript Title: Different types of dietary fibers trigger specific alterations in composition and predicted functions of colonic bacterial communities in BALB/c mice

The names of the authors: Yuheng Luo[1†*], Ling Zhang[1†], Hua Li[1†], Hauke Smidt[2], André Denis G. Wright[3], Keying Zhang[1], Xuemei Ding[1], Qiufeng Zeng[1], Shiping Bai[1], Jianping Wang[1], Jian Li[1], Ping Zheng[1], Gang Tian[1], Jingyi Cai[1], Daiwen Chen[1]

The affiliations and addresses of the authors: [1]Key Laboratory for Animal Disease-Resistance Nutrition of China, Ministry of Education, Animal Nutrition Institute, Sichuan Agricultural University, Chengdu, China; [2]School of Animal and Comparative Biomedical Sciences, University of Arizona, 1117 E. Lowell Street, Tucson, AZ 85721, USA; [3]Laboratory of Microbiology, Wageningen University, Dreijenplein 10, 6703 HB Wageningen, The Netherlands

[†]Both authors contributed equally to this work.

[*]For correspondence. Dr.Yu-heng Luo, fax 00 86 28 86290922 and tel 00 86 28 82690976, email luoluo212@126.com

Table S1 Macronutrient composition of experimental diets

	C	G	M	GM
**Diet(g/kg)**				
Casein	233	200	233	212
Corn starch	316	280	316	280
Sucrose	60	60	60	83
Palm kernel fat	111	100	111	105
bean oil	10	10	10	10
Linseed oil	10	10	10	10
Oat-derived β-glucan	0	280	0	140
MCC	0	0	200	100
bentonite clay	200	0	0	0
Mineral mixture[1]	50	50	50	50
Vitamin mixture[2]	10	10	10	10
Total amount of diet(g)	1000	1000	1000	1000
**Calculated macronutrient metabolizable energy(%)**				
Protein	25.51	20.00	21.41	19.98
Carbohydrates	46.22	55.49	54.88	56.02
Fat	28.27	24.50	23.72	23.99
**Measured energy and protein**				
Energy (Cal/g)	4130.74	4307.28	4291.75	4204.88
Protein (%)	17.62	16.45	16.72	16.65
Energy (Cal/g)/Protein (%)	234.44	261.85	256.61	252.60

[1]Mineralswere added to the diets in the form of AIN-93G-MX mineral mix

[2]Vitaminswere added to the diets in the form of AIN-93-VX vitamin mix

C, control; G, glucan; M, MCC; GM, glucan and MCC.

As the β-glucan is extracted from oat, the remaining 29% is mainly composed by small amount of oat bran and protein. Thus only 70% of oat-β-glucan was considered as carbohydrate, and the left 30% was considered as protein which was calculated according to the proportion of protein in oat bran. In current study, not only total protein and carbohydrate, but also the balance of total energy between groups was considered. As the amount of the dietary fibers was too large (approximately 20%), total energy of diet of the four groups can only be calculated as closer as possible. Therefore, the total energy of feed in control diet was reduced by the supplement of bentoniteclay which is always used in swine feed for energy regulation as its inertcharacteristic.Alternatively, the measured energy (Cal/g) / protein (%) for each diet remained no significant change ($p > 0.05$).

Table S2 OTU table summary

Sample ID	Sequence counts	Observations (OTU number)
C1	109,010	943
C2	91,608	887
C3	88,607	950
C4	78,438	791
C5	54,282	972
C6	76,028	988
C7	77,422	966
G1	86,313	591
G2	83,659	699
G3	92,349	467
G4	90,202	562
G5	69,899	511
G6	56,753	443
G7	78,127	570
M1	101,396	871
M2	79,105	789
M3	74,422	692
M4	73,145	797
M5	86,767	1,287
M6	94,271	982
M7	102,035	926
GM1	83,068	560
GM2	79,849	726
GM3	98,640	676
GM4	86,321	681
GM5	81,901	708
GM6	69,042	573
GM7	53,366	462

C, control; G, glucan; M, MCC; GM, glucan and MCC.

Table S3 The relative abundance of main bacterial taxa in the colon of mice in different treatments (%)

Taxon	C	G	M	GM	SD-C	SD-G	SD-M	SD-GM	*p*-value
[*Prevotella*]§	0.141	0.730	0.251	0.390	0.34	0.62	0.29	0.62	0.16
**[*Ruminococcus*]***	1.170	0.457	0.787	0.458	0.68	0.33	0.40	0.33	**0.02**
AF12	0.201	0.036	0.289	0.283	0.22	0.07	0.31	0.35	0.25
Anaeroplasma	0.249	0.247	0.119	0.615	0.28	0.58	0.19	1.56	0.72
Bacteroidales	1.456	6.062	3.214	1.461	0.82	9.76	2.88	1.24	0.31
Bacteroides**	0.337	6.544	0.824	1.262	0.21	6.84	0.48	1.26	**0.01**
Christensenellaceae	0.067	0.098	0.210	0.068	0.06	0.19	0.30	0.16	0.50
Clostridiales**	50.879	21.071	37.330	35.512	5.41	10.99	9.84	10.01	**0.00**
Clostridiales*.Other.Other	0.330	0.092	0.058	0.003	0.38	0.14	0.10	0.00	**0.03**
Clostridium§	0.057	0.150	0.028	0.013	0.06	0.17	0.06	0.03	0.07
Coprococcus**	0.856	0.301	0.859	0.355	0.49	0.21	0.40	0.31	**0.01**
Dechloromonas	0.031	0.111	0.029	0.032	0.03	0.14	0.05	0.06	0.20
Desulfovibrio§	0.953	0.251	1.184	1.387	0.48	0.17	0.78	1.63	0.15
Desulfovibrionaceae**	1.393	0.473	1.678	0.623	0.91	0.42	0.65	0.59	**0.01**
Dorea	0.124	0.180	0.054	0.095	0.15	0.17	0.05	0.07	0.30
Enterobacteriaceae*	0.230	1.772	0.353	0.297	0.20	1.86	0.50	0.43	**0.02**
Erysipelotrichaceae**	0.126	0.024	0.025	0.050	0.08	0.03	0.03	0.06	**0.01**
Flexispira	1.166	0.528	0.505	1.015	1.51	0.43	1.03	1.75	0.70
Helicobacter	0.152	0.093	0.099	0.210	0.22	0.12	0.11	0.32	0.71
Helicobacteraceae§	0.148	0.820	0.422	1.043	0.14	0.75	0.40	0.93	0.06
Helicobacteraceae.Other	0.233	0.103	0.000	0.000	0.62	0.27	0.00	0.00	0.53
Lachnospiraceae§	14.221	7.723	10.685	10.079	3.82	5.78	4.24	3.50	0.08
Lactobacillus	0.116	0.196	0.199	0.128	0.15	0.14	0.21	0.10	0.65
Mucispirillum	1.229	0.232	0.751	1.320	0.88	0.21	1.20	1.47	0.22
Odoribacter§	1.747	0.803	2.581	2.207	0.79	0.55	1.70	1.95	0.12
Oscillospira§	3.828	2.842	4.398	5.737	1.62	0.95	1.31	3.07	0.06
Parabacteroides	0.076	0.000	0.074	0.199	0.14	0.00	0.13	0.52	0.62
Prevotella*	0.099	1.380	0.251	0.294	0.08	1.52	0.20	0.38	**0.02**
Pseudomonadaceae	0.098	0.068	0.147	0.128	0.06	0.06	0.15	0.15	0.59
Pseudomonas	0.092	0.038	0.083	0.102	0.13	0.03	0.05	0.13	0.63
Rikenella**	0.867	0.114	0.345	0.117	0.61	0.15	0.38	0.12	**0.00**
Rikenellaceae*	3.823	1.587	6.471	3.559	1.91	0.74	3.39	3.11	**0.01**
Ruminococcaceae§	5.500	3.638	7.065	5.122	1.20	2.68	3.97	3.36	0.23
Ruminococcaceae.Other§	0.295	0.045	0.447	0.473	0.15	0.03	0.52	0.44	0.11
Ruminococcus§	2.116	1.475	1.542	3.992	0.74	0.76	0.55	3.72	0.08
**S24-7****	4.108	38.073	15.002	20.180	1.97	17.06	8.47	13.86	**0.00**
Sutterella**	0.000	0.149	0.000	0.015	0.00	0.16	0.00	0.02	**0.00**

The difference on abundance of main bacterial taxa among the four groups was analyzed with One-Way ANOVA using SPSS 16.0. Only taxa that had a relative abundance of 0.01% in at least one of the treatments were shown and stated. The cutoff 0.01% was only for data in Table S3 but not for data shown in other tables or figures. The color depth of back ground of each cell based on red-yellow-green color gradation shows the abundance of each taxon. SD, standard deviation; C,

control; G, glucan; M, MCC; GM, glucan and MCC.
Differences were considered significant when $p < 0.05$. *$p < 0.05$; **$p < 0.01$; § Although the p-value showed no significant difference, at least two treatments showed significantly different.

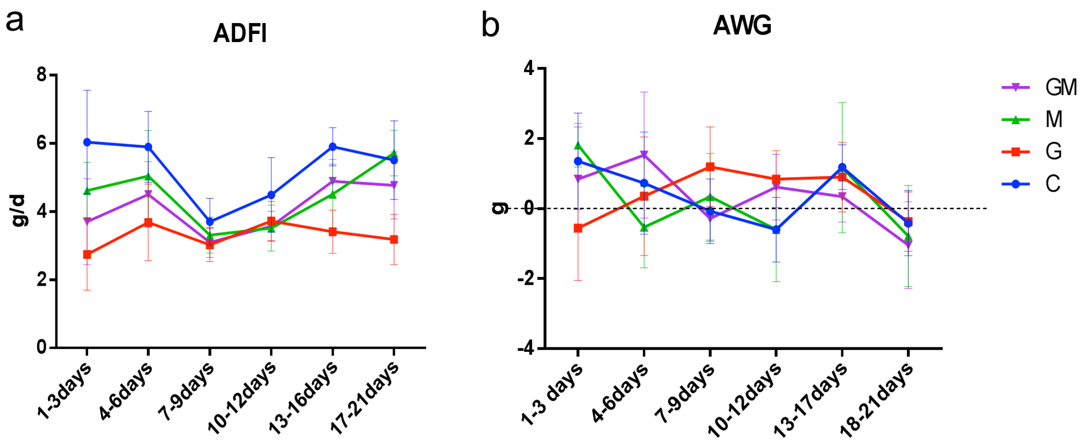

Figure S1 Change of ADFI (a) and AWG (b) of mice in the four groups. C, control group; G, mice fed with oat-derived β-glucan; M mice fed with MCC; GM mice fed with the mixture of the two dietary fibers.

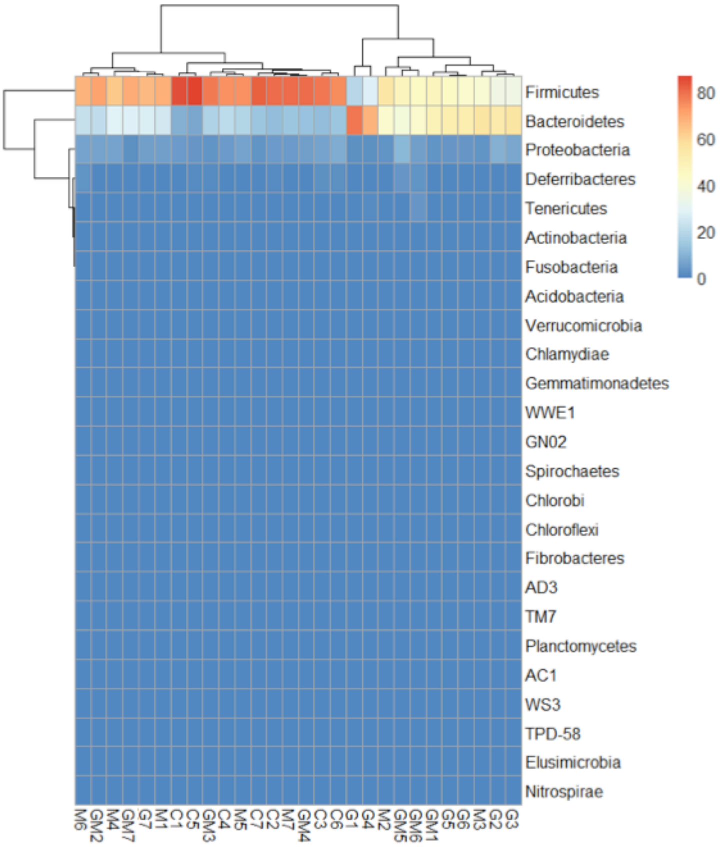

Figure S2 Heatmap of bacterial phyla in the colonic samples. Phylaarearranged in rows and are clusteredon the vertical axis (y-axis).Samples arearranged vertically and are clustered on thehorizontal axis (x-axis).Clustering was done using Phylotrac's heatmap optionwith Pearson correlations andcomplete lineage algorithms.Different colors indicate therelative abundance of the different phyla.C, control group; G, mice fed with oat-derived β-glucan; M mice fed with MCC; GM mice fed with the mixture of the two dietary fibers.

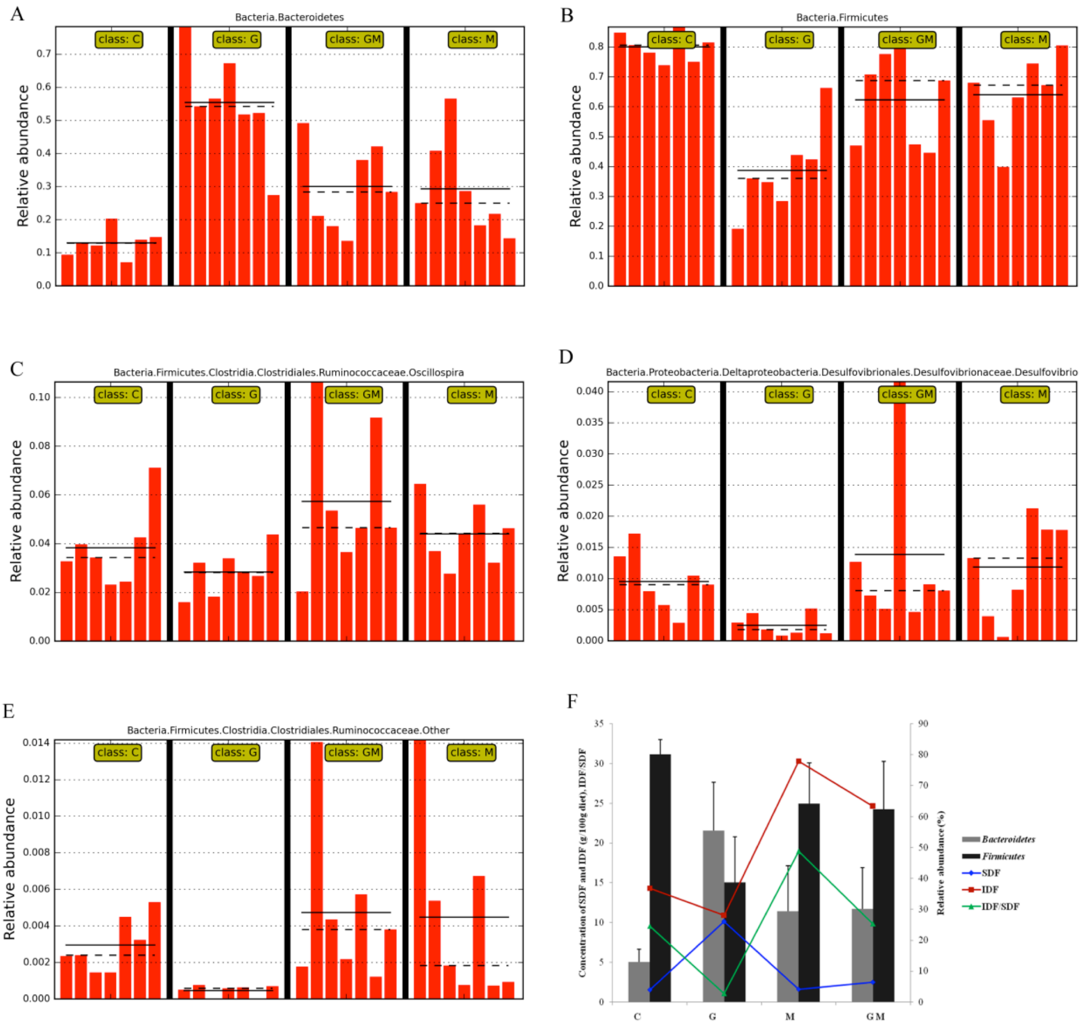

Figure S3 Relative abundance of several bacterial phyla and genera in the colonic samples. (A) *Bacteroidetes*, (B) *Firmicutes*, (C) *Oscillospira* spp., (D) *Desulfovibrio* spp., (E) *Ruminoccaceae*, (F) the change of *Bacteroidetes* and *Firmicutes*, and their correlation with dietary SDF, IDF concentration and IDF/SDF ratio. C, control; G, glucan; M, MCC; GM, glucan and MCC.

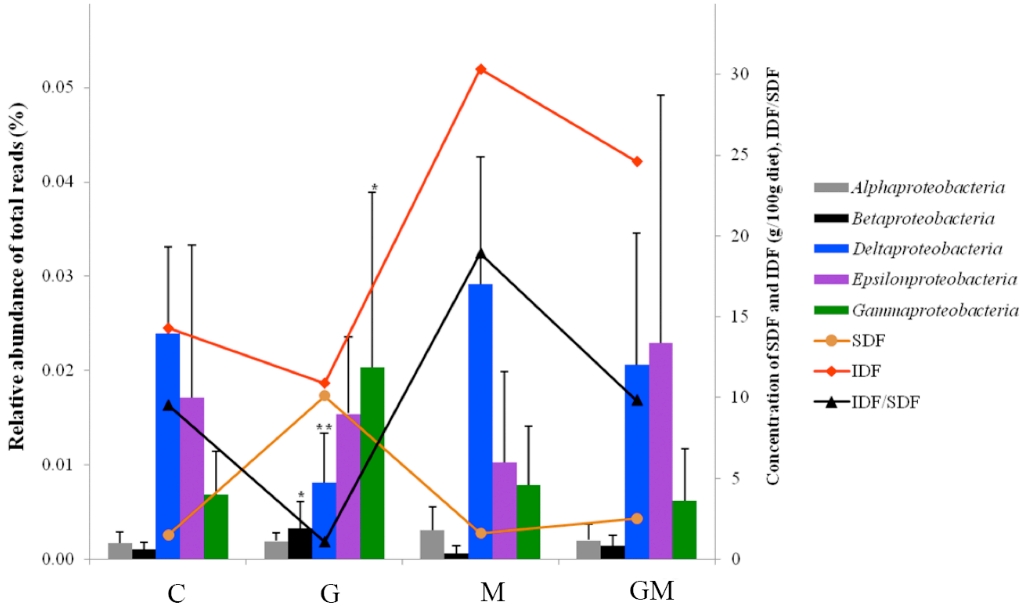

Figure S4 Relative abundance of five classes of *Proteobacteria* in the four treatments. Broken lines show the change of dietary SDF and IDF concentration and IDF/SDF ratio in the four treatments. C, control; G, glucan; M, MCC; GM, glucan and MCC.

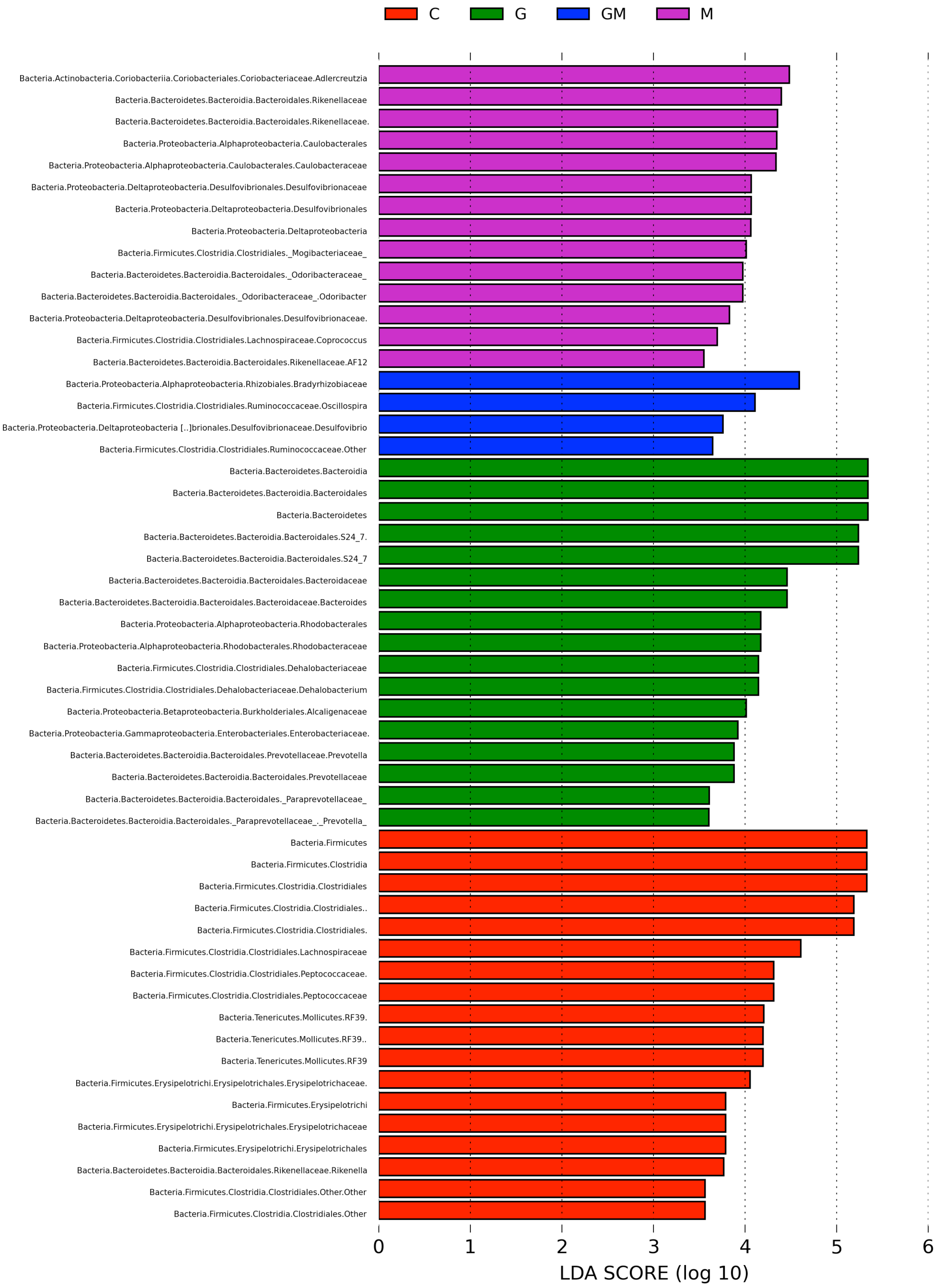

Figure S5. Bacterial taxa significantly different between the four treatments as identified by linear discriminant analysis coupled with effect size (LEfSe) using default parameters.

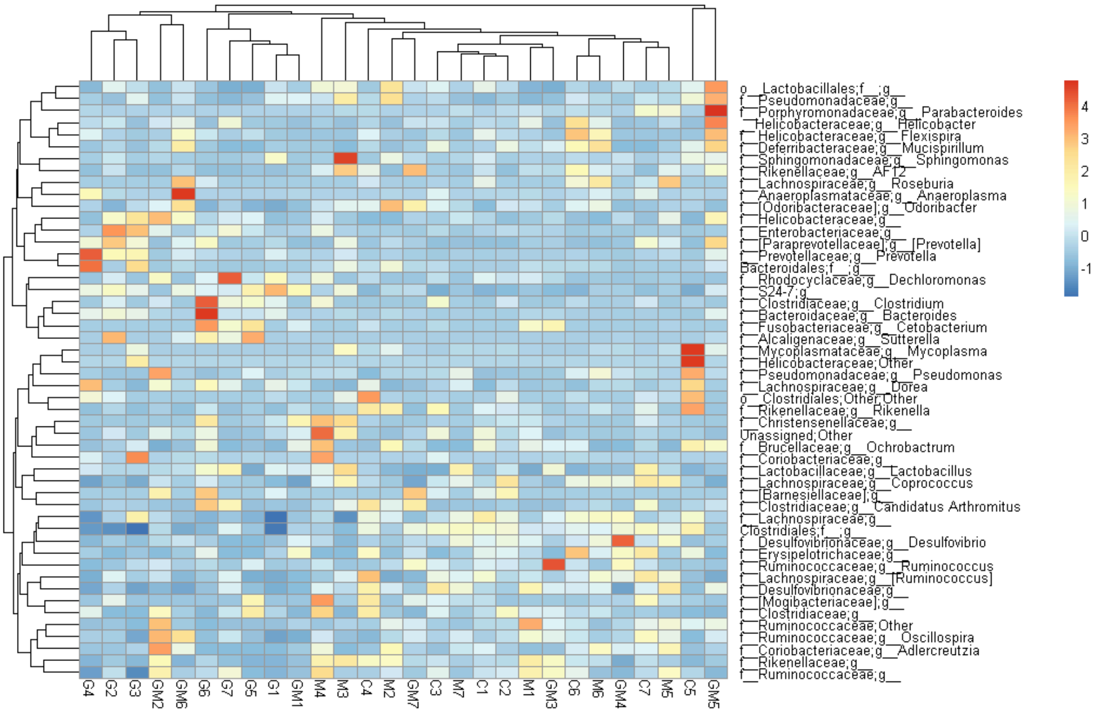

Figure S6 Heatmap of top 50 bacterial genera in the colonic samples. OTUs arearranged in rows and are clusteredon the vertical axis (y-axis).Samples arearranged vertically and are on thehorizontal axis (x-axis).Clustering was done for eachusing Phylotrac’s heatmap optionwith Pearson correlations andcomplete lineage algorithms.Different colors indicate therelative abundance of the taxons.C control group, G mice fed with oat-derived â-glucan, M mice fed with MCC, GM mice fed with the mixture of the two dietary fibers.

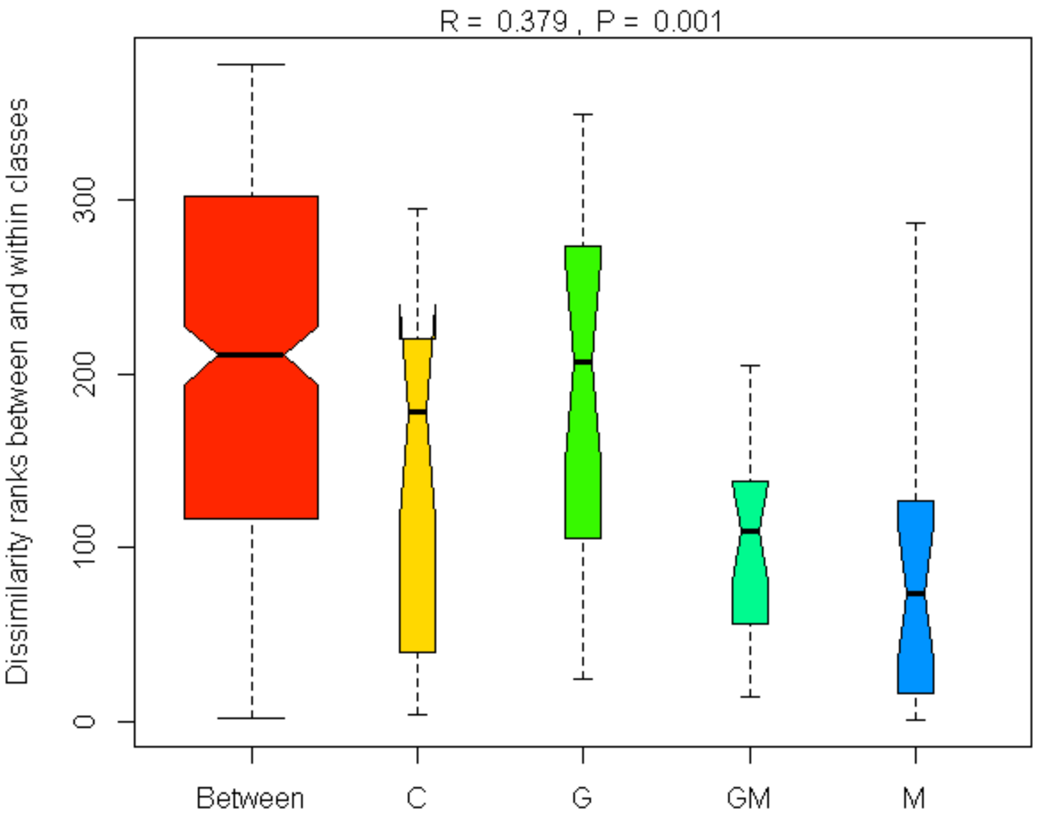

Figure S7 Analysis of similarities (ANOSIM) of bacterial diversity of the four groups. ANOSIM statistic R is 0.379, $p = 0.001$. C control group, G mice fed with oat-derived â-glucan, M mice fed with MCC, GM mice fed with the mixture of the two dietary fibers.
